# Prevalence of vitamin D deficiency and its association with cardiometabolic risk factors among healthcare workers in the Eastern Cape province, South Africa; cross-sectional study

**DOI:** 10.1038/s41598-024-54977-y

**Published:** 2024-02-27

**Authors:** Oladele Vincent Adeniyi, Charity Masilela, Jaya A. George

**Affiliations:** 1 Department of Family Medicine, Walter Sisulu University, 5219, East London, South Africa; 2 Department of Biochemistry and Microbiology, University of Zululand, KwaDlangezwa, South Africa; 3 Department of Chemical Pathology, National Health Laboratory Service and University of the Witwatersrand, Parktown, Johannesburg South Africa

**Keywords:** Cardiometabolic risk factors, Eastern Cape, Healthcare workers, South Africa, Vitamin D deficiency, Endocrinology, Medical research

## Abstract

This study assesses the prevalence of Vitamin D deficiency and its potential association with cardiometabolic risk factors among South African adults residing in the Eastern Cape province. In this cross-sectional study, 1244 healthcare workers (HCWs) completed a self-administered questionnaire and venous blood samples were drawn at two academic hospitals in the Eastern Cape. History of hypertension and diabetes mellitus were self-reported. Participants were categorised as obese if their body mass index (BMI) ≥ 30 kg/m^2^. Participants were classified as having metabolic syndrome if they had hypertension, diabetes mellitus and obesity. Vitamin D [25(OH)D] deficiency was defined as venous blood concentrations < 50 nmol/L. Associations between vitamin D deficiency and participants’ characteristics were assessed using multivariate logistic regression model analysis. The prevalence of vitamin D deficiency was 28.5% (n = 355), of whom 292 were female. Among the participants who were deficient in vitamin D, the prevalence of obesity, diabetes mellitus, hypertension, chronic kidney disease, and metabolic syndrome was 64.9% (n = 230), 9% (n = 32), 16.6% (n = 59), 2.3% (n = 8) and 18% (n = 64), respectively. In the adjusted multivariate logistic regression model, black Africans (AOR = 2.87; 95% CI 1.52–5.43) and individuals ≥ 42 years (AOR = 1.37; 95% CI 1.07–1.77) were more likely to exhibit vitamin D deficiency. However, there was no significant association by age, sex, and cardiometabolic markers. More than one in four healthcare workers was deficient in vitamin D among the study sample, especially the black Africans and older individuals. Further studies are needed at the population level to elucidate on the vitamin D status in the region.

## Introduction

Cardiometabolic disorders represent a cluster of interrelated risk factors, including hypertension, diabetes mellitus (DM), dyslipidemia and obesity^1–3^. These cardiometabolic risk factors accelerate the progression of cardiovascular diseases (CVDs)^3^, which account for 17.9 million deaths annually. According to the World Health Organisation (WHO), over three quarters of CVD-associated deaths occur in low- and middle-income countries^4^. South Africa is no exception; it has been reported that 215 people in South Africa die every day from heart disease or stroke^4^. It was further demonstrated that the age standardised death rate from non-communicable diseases (NCDs) among South Africans is higher than that of tuberculosis and HIV/AIDS combined, with CVDs in the leading category of NCDs^5^. In recent years, vitamin D deficiency has been implicated in the pathophysiology of CVDs and has been shown to be associated with cardiometabolic risk factors, including chronic low-grade inflammation, obesity and DM among individuals of different ethnicities^6–8^. Thus, treating vitamin D deficiency may offer a feasible and cost-effective means of reducing cardiometabolic risk factors at the population level.

Vitamin D is a group of fat-soluble steroids that can be obtained from diet, supplements, and conversion of 7-dehydrocholesterol in the lower layers of the epidermis by ultraviolet B (UVB) radiation^9^. In the liver, Vitamin D is hydroxylated to 25-hydroxyvitamin D (25[OH]D), the major circulating vitamin D metabolite, and 25[OH]D is further metabolised to its metabolically active form, 1,25-dihydroxyvitamin D (1,25[OH]2D), by 1α-hydroxylase^9–11^. Furthermore, vitamin D is known for its role in the absorption of several minerals including calcium^9,11^. Calcium plays an important role in cellular signal transduction, protein synthesis, bone formation and maintaining potential differences across excitable cell membranes. This makes vitamin D an important indicator of the status of bone health, certain types of cancer and multiple autoimmune diseases^12,13^.

Vitamin D deficiency has been documented across different age groups and ethnicities^11,14–17^. Owing to the increasing prevalence and associated health risks, vitamin D deficiency has become a major public health issue worldwide^18^. Experimental studies suggest that vitamin D plays an important role in increasing the risk of cardiometabolic diseases through multiple pathways, including the inhibition of the release of proinflammatory cytokines and regulation of the renin-angiotensin system^19^. Human observational studies have shown that low dietary vitamin D intake is inversely related to the various cardiometabolic risk factors^14,20–22^. Evidence from randomised clinical trials for the effect of vitamin D supplements on cardiometabolic risk factors, however, remains inconsistent^22,23^.

In addition, most studies have been conducted in Western countries and in more affluent provinces in South Africa^22^, where the prevalence of vitamin D deficiency ranges from 7 to 62.7%^15,24–26^. There is thus a gap in knowledge about the prevalence of vitamin D deficiency among the adult population of the Eastern Cape province in South Africa, a generally poor province. In addition, there is little evidence supporting the association of known cardiometabolic risk factors with Vitamin D deficiency across different population groups in South Africa. The current study bridges this knowledge gap by reporting on the prevalence of Vitamin D deficiency and its potential association with cardiometabolic risk factors among South African adults residing in the Eastern Cape province.

## Methods

### Ethical approval and consent to participate

The larger study received approval from the Walter Sisulu University Ethics Committee (WSU/087/2020), followed by additional approval from the Cecilia Makiwane and Frere Hospitals Research Ethics Committee to allow the measurement of vitamin D levels and monitor reinfection rate (FCMHREC/A068/2020). Participants gave written informed consent for their voluntary participation in the study. Participants’ right to privacy and confidentiality of medical information were respected during and after the study’s completion. The study followed the Helsinki Declaration and Good Clinical Practice Guidelines as well as local institutional policies on human research.

### Study design, settings and population

This observational cross-sectional study forms part of a larger study which aimed at assessing the cumulative incidence of severe acute respiratory syndrome coronavirus-2 (SARS-CoV-2) infections among healthcare workers (HCWs) in the Eastern Cape. The detailed methodology has been published previously^27^. Briefly, healthcare workers working at the two academic hospitals (Frere and Cecilia Makiwane) in the central region of the Eastern Cape province participated in the study from November 2020 to February 2021.

Prior to recruitment, an awareness campaign was carried out through the union leaders, departmental heads and unit managers. In addition, a communique detailing the purpose, process and expected outcomes of the study was circulated among the HCWs in the two hospitals. Each working area was allocated specific days for data capturing. In addition, a central area was created to allow for staff who may have missed their departmental allotted days to catch up. There was no sample size estimated for the study as provision was made to recruit as many staff within the time frame of the study. All categories of healthcare workers (clinical and non-clinical staff) were eligible to take part in the study. The study adopted a multi-stage sampling technique in recruiting participants across all work areas in the two hospitals^27^.

### Study procedure

Two research nurses and four assistants received training on the study process and its implementation. The nurses drew 5 millilitres of venous blood samples from each participant using an aseptic technique. Participants’ height was measured to the nearest 0.1 cm with a mounted stadiometer while their weights were measured with light clothing to the nearest 0.1 kg with a digital scale (Tanita-HD 309, Creative Health Products, MI, USA). The body mass index (BMI) was estimated as the ratio of weight in kilogrammes (kg) to height in metres squared (m^2^). Each participant completed the self-administered questionnaire. Each was issued a unique identifier code to encode the name, date of birth and work area, and also, to allow linkage with blood results while maintaining privacy and confidentiality of medical information. Responses to the questionnaire data were captured in the REDCap database of the South African Medical Research Council Server.

### Measures

#### Laboratory assessment

Laboratory analysis of plasma samples for vitamin D followed standard protocols^25^. Briefly, samples for vitamin D were obtained from freshly collected venous samples and were stored at – 80 °C until laboratory analysis. We performed a short liquid–liquid extraction prior to measuring the concentration of 25(OH)D by using high performance liquid chromatography (HPLC) kit (Recipe, München, Germany) with a photodiode array (PDA) detector. Thereafter, aliquots of plasma samples were injected into the HPLC system, and the analytes were separated on the appropriate analytical column. At a wavelength of 254 nm, both 25(OH)D_2_ and 25(OH)D_3_ were detected and separated. The chromatograms were integrated using the peak height. The sum of 25(OH)D_2_ and 25(OH)D_3_ was estimated as the total vitamin D [25(OH)D level in each participant. The limit of detection of assay for 25(OH)D_3_ was 2.5 nmol/L and 7.5 nmol/L for 25(OH)D_2_ while the intra-assay CV for 25(OH)D ranged from 0.9 to 4.9%, and the inter-assay CV ranged from 3.0 to 4.9%. Laboratory analysis was performed by the National Health Laboratory Service, which fulfilled the requirements of the mandatory vitamin D external quality assurance scheme (DEQAS). Vitamin D [25(OH)D was categorised as deficient and insufficient if concentrations were less than 50 nmol/L and 50–75 nmol/L, respectively^28,29^.

### Covariates

Relevant sociodemographic and clinical covariates were included in this study. For the multivariate analysis, participants were categorised by median age to those < 42 years and those ≥ 42 years, place of residence was categorised as suburban and urban, and ethnicity was categorised as black (African ancestry), white (Caucasian) or other (Asians and mixed ancestry), all self-reported in the questionnaire. Established cardiometabolic risk factors such as obesity (defined as body mass index (BMI) ≥ 30 kg/m^2^) was estimated as ratio of weight and height squared while prior diagnosis of diabetes, hypertension and chronic kidney disease were self-reported in the questionnaire. BMI was further categorised into underweight = BMI < 18.5 kg/m^2^; normal weight = BMI: 18.5–24.9 kg/m^2^ and overweight = BMI: 25.0–29.9 kg/m^2^. Participants were classified as having metabolic syndrome if they met the harmonised criteria of having all three conditions: hypertension, diabetes mellitus and obesity^30^.

### Statistical analysis

Complete data for 1244 participants was captured on the Research Electronic Data Capture (Redcap) and analysed using the IBM SPSS Statistics for Windows, Version 27.0 (IBM Corp., Armonk, New York, USA). The characteristics of the study participants in frequencies and percentages were reported for categorical variables. Differences between the means of continuous variables were assessed using an independent sample t-test, and relationships between vitamin D levels and the categorical variables were assessed using chi-square analysis. Associations between the main outcome measure (vitamin D deficiency < 50 nmol/L) and participants’ characteristics were assessed using multivariate logistic regression model analysis (both crude and adjusted odds ratios) with a 95% confidence interval (95% CI). Age, sex, ethnicity, level of education, smoking status, residence and residence and SARS-CoV-2 Positive test were used as the main adjusting factors. A p-value of less than 0.05 was considered statistically significant. Furthermore, Pearson chi-square analysis (Goodness-of-Fit) was used to assess how well the model fits the data. Large chi-square values were an indication of a poor fit for the model, and statistically significant values (i.e., p < 0.05) indicated that the model did not fit the data well.

## Results

### General characteristics

A total of 1244 individuals participated in this study, of whom 18.3% (n = 227) were male and 81.8% were female (n = 1017). Furthermore, 87.5% (n = 1088) of the participants were black, 8.6% (n = 107) were white and 3.9% (n = 49) belonged to other ethnicities. About 63.1% (n = 781) were obese, 57.6% (n = 716) were resident in suburban areas, and 18.4% met the criteria for metabolic syndrome (n = 229). Furthermore, the mean age of the study participants was 42.6 ± 11.1 years, with similar patterns by sex and ethnicity. The mean BMI and vitamin D level for the general study population was 32 ± 7.7 kg/m^2^ and 63.2 ± 24.3 nmol/L, respectively. However, the mean circulating vitamin D level was higher among the women (68.4 ± 14.5) nmol/L and white participants (75.5 ± 32.7). Additionally, an independent t-test was run to assess the mean differences between the continuous variables (age, BMI, and Vitamin D levels) against sex and ethnicity. The data revealed that only vitamin D levels significantly differed across the three ethnic groups (p < 0.001) while the other variables did not reach significance level (Table 1).

**Table 1:** General characteristics of the study participants stratified by sex and ethnicity (n = 1244).

Variable	Total	Sex		Ethnicity		
		Male	Female	Black	White	Other
All	1244 (100)	227 (18.2)	1017 (81.8)	1088 (87.5)	107 (8.6)	49 (3.9)
*Age (years)	42.6 ± 11.1	41.6 ± 10.9	42.8 ± 11.3	42.9 ± 10.8	41.7 ± 12.4	41.1 ± 11.9
*Vitamin D (nmol/L)	63.2 ± 24.3	60.4 ± 235	68.4 ± 14.5	62.7 ± 23.9	75.5 ± 32.7	60.6 ± 25.4
BMI (kg/m^2^)	32 ± 7.7	28.8 ± 5.6	33.9 ± 7.9	34.1 ± 7.1***	28.0 ± 6.7***	29.4 ± 7.0***
Age (Years)						
18–25	71 (5.7)	15 (6.6)	56 (5.5)	41 (3.8)	20 (18.7)	10 (20.4)
26–35	308 (24.8)	72 (31.7)	236 (23.2)	249 (22.9)	44 (41.1)	15 (30.6)
36–45	336 (27.0)	72 (31.7)	264 (26.0)	311 (28.6)	12 (11.2)	13 (26.5)
46–55	332 (26.7)	49 (21.6)	283 (27.8)	305 (28.0)	20 (18.7)	07 (14.3)
56–65	197 (15.8)	19 (8.4)	178 (17.5)	182 (16.7)	11 (10.3)	04 (8.2)
Smoking status						
Never	1136 (91.3)	172 (75.8)	964 (94.8)	1004 (92.3)	91 (85.0)	41 (83.7)
Active	66 (5.3)	32 (14.1)	34 (3.3)	52 (4.8)	10 (9.3)	04 (8.2)
Former	42 (3.4)	23 (10.1)	19 (1.9)	32 (2.9)	06 (5.6)	04 (8.2)
Level of education						
Secondary	360 (28.9)	62 (27.3)	298 (29.3)	346 (31.8)	12 (11.2)	02 (4.1)
Tertiary	884 (71.1)	165 (72.7)	719 (70.7)	742 (68.2)	95 (88.8)	47 (95.9)
BMI						
Underweight	7 (0.6)	2 (0.9)	5 (0.5)	5 (0.5)	02 (1.9)	0 (0.0)
Normal	171 (13.8)	48 (21.4)	123 (12.1)	109 (10.1)	42 (39.6)	20 (40.8)
Overweight	279 (22.5)	82 (36.6)	197 (19.4)	234 (21.6)	29 (27.4)	16 (32.7)
Obese	781 (63.1)	92 (41.1)	689 (67.9)	735 (67.9)	33 (31.1)	13 (26.5)
Residence						
Urban	528 (42.44)	89 (39.2)	439 (43.2)	523 (48.1)	02 (1.9)	03 (6.1)
Suburban	716 (57.6)	138 (60.8)	578 (56.8)	565 (51.9)	105 (98.1)	46 (93.9)
Diabetes						
Yes	98 (7.9)	14 (6.2)	84 (8.3)	91 (8.4)	5 (4.7)	02 (4.1)
No	1146 (92.1)	213 (93.8)	933 (91.7)	997 (91.6)	102 (95.3)	47 (95.9)
Hypertension						
Yes	227 (18.2)	27 (11.9)	200 (19.7)	205 (18.8)	16 (15.0)	06 (12.2)
No	1017 (81.8)	200 (88.1)	817 (80.3)	883 (81.2)	91 (85.0)	43 (87.8)
CKD						
Yes	24 (1.9)	03 (1.3)	21 2.1 (2.1)	23 (2.1)	01 (0.9)	0 (0.0)
No	1220 (98.1)	224 (98.7)	996 (97.9)	1065 (97.9)	106 (99.1)	49 (100.0)
Positive SARS-CoV-2 test						
Yes	388 (31.2)	59 (26.0)	329 (32.4)	350 (32.2)	24 (22.4)	14 (28.6)
No	856 (68.8)	168 (74.0)	688 (67.6)	738 (67.8)	83 (77.6)	35 (71.4)
Metabolic syndrome						
Yes	229 (18.4)	24 (10.6)	205 (20.2)	211 (19.4)	15 (14.0)	03 (6.1)
No	1015 (81.6)	203 (89.4)	812 (79.8)	877 (80.6)	92 (86.0)	46 (93.9)
Vitamin D status (nmol/L)						
Deficient (< 50)	355 (28.5)	63 (27.8)	292 (28.7)	324 (29.8)	06 (5.6)	25 (51.0)
Insufficient (50–75)	536 (43.1)	95 (41.9)	441 (43.4)	480 (44.1)	44 (41.1)	12 (24.5)
Sufficient (> 75)	353 (28.4)	69 (30.4)	284 (27.9)	284 (26.1)	57 (53.3)	12 (24.5)

Values are reported as mean (standard deviation)* and n (%)**.

*BMI* body mass index, *CKD* chronic kidney disease.

***p < 0.001**.**

### Prevalence of vitamin D deficiency and cardiometabolic risk factors

Overall, the prevalence of vitamin D deficiency was 28.5% (95% CI 0.26–0.31) and insufficiency was 43.1% (95% CI 0.40–0.46). Though there was a higher prevalence of vitamin D deficiency among women 28.7% (n = 292) in comparison to 27.8% in men (n = 63), the difference was not statistically significant (p > 0.05). The overall prevalence of obesity (BMI ≥ 30 kg/m^2^), DM, hypertension, CKD and metabolic syndrome was 63.1% (n = 781), 7.9% (n = 98), 18.2% (n = 227), 1.9% (n = 24) and 18.4% (n = 229), respectively. Among participants who met the criteria for vitamin D deficiency, the prevalence of obesity, DM, hypertension, CKD and metabolic syndrome was 64.9% (n = 230/355), 9% (n = 32/355), 16.6% (n = 59/355), 2.3% (n = 8/355) and 18% (n = 64/355), respectively.

### Determinants of vitamin D deficiency

In the chi-square analysis, vitamin D status showed a significant relationship with age (p = 0.026) and ethnicity (p < 0.001). Almost 30% of black African healthcare workers were deficient in vitamin D in comparison to 5.6% among the white participants. However, a higher proportion of mixed ancestry and Asian (other) population (51%) were deficient in vitamin D in comparison to the other two groups (Black and White participants). On the other hand, no relationship was observed between vitamin D status and the other selected variables (sex, residence, level of education, smoking status, positive SARS-CoV-2 test, BMI, DM, hypertension, CKD and metabolic syndrome).

The multivariate logistic regression (unadjusted) analysis showed that black participants (COR = 7.18; 95% CI 3.11–16.55) and participants ≥ 42 years (COR = 1.35; 95% CI 1.03–1.69) were more likely to exhibit vitamin D deficiency, while white participants (COR = 0.41; 95% CI 0.23–0.72) were less likely to exhibit vitamin D deficiency.

After adjusting for confounding factors, the final multivariate logistic regression analysis shows that black participants (AOR = 2.87 95% CI 1.52–5.43) were more likely to exhibit vitamin D deficiency. Similarly, participants who were ≥ 42 years (AOR = 1.37; 95% CI 1.07–1.77) were more likely to exhibit vitamin D deficiency. None of the other variables was significantly associated with vitamin D status. Different models (Model I–V) were performed and all confirmed aging and black ethnicity were the independent determinants of vitamin D deficiency in the study sample. Additionally, the significance levels of each model are shown in Table 2.

**Table 2:** Multivariate logistic regression analysis of factors associated with Vitamin D deficiency.

Variable	Model I	Model II	Model III	Model IV	Model V
Sex					
Male	1	1	1	1	1
Female	1.02 (0.72–1.44)	1.01 (0.72–1.42)	1.01 (0.72–141)	1.02 (0.73–1.43)	1.01 (0.72–1.41)
Age (Years)					
< 42	1	1	1	1	1
≥ 42	1.36 (1.05–1.75)*	1.36 (1.06–1.75) *	1.37 (1.06–1.76)*	1.36 (1.06–1.75)*	1.36 (1.06–1.75)*
**Ethnicity					
Other	1	1	1	1	1
Black	2.84 (1.51–5.37)**	2.84 (1.51–5.35)***	2.85 (1.51–5.37)**	2.85 (1.51–5.37)***	2.28 (1.50–5.41)**
White	1.26 (0.86–1.86)	1.22 (0.82–1.80)	1.22 (0.83–1.80)	1.23 (0.84–1.81)	1.22 (0.83–1.79)
Residence					
Urban	1	1	1	1	1
Suburban	1.00 (0.76–1.31)	0.98 (0.75–1.29)	0.99 (0.76–1.41)	0.97 (0.74–1.24)	0.99 (0.75–1.29)
Smoking status					
Active	1		1	1	1
Former	0.84 (0.38–1.16)	1	0.77 (0.36–1.64)	0.78 (0.36–1.64)	0.78 (0.37–1.66)
Never	1.13 (0.44–2.85)	1.15 (0.86–1.54)	1.10 (0.43–2.80)	1.11 (0.44–2.82)	1.12 (0.44–2.84)
Level of education					
Secondary	1	1	1	1	1
Tertiary	1.17 (0.87–1.56)	1.15 (0.86–1.54)	1.15 (0.86–1.54)	1.15 (0.86–1.53)	1.60 (0.87–1.54)
SARS-Cov-2 positive test					
No	1	1	1	1	1
Yes	1.06 (0.80–1.39)	1.04 (0.80–1.37)	1.05 (0.80–1.38)	1.49 (0.79–1.36)	1.04 (0.80–1.37)
Obesity		–	–	–	–
Non-obese	1				
Obese	0.89 (0.68–1.18)				
Diabetes	–	–		–	–
No			1		
Yes			0.78 (0.50–1.23)		
Hypertension	–	–	–		–
No				1	
Yes				1.73 (0.84–1.63)	
CKD	–	–	–	–	
No					1
Yes					0.82 (0.36–2.07)
Metabolic syndrome	–		–	–	–
No		1			
Yes		1.30 (0.74–1.42)			
p-values	0.173	0.177	0.276	0.182	0.132

Significant values are in bold.

Values are reported as odds ratios (95%CI); *CKD* chronic kidney disease; Model I = Obesity, adjusted by age, sex and ethnicity, level of education, smoking status, residence and SARS-CoV-2 Positive test; Model II = Metabolic syndrome, adjusted by age, sex and ethnicity, level of education, smoking status, residence and SARS-CoV-2 Positive test. Model III = Diabetes, adjusted by age, sex and ethnicity, level of education, smoking status, residence and SARS-CoV-2 Positive test; Model IV = Hypertension, adjusted by age, sex and ethnicity, level of education, smoking status, residence and SARS-CoV-2 Positive test; Model V = CKD, adjusted by age, sex and ethnicity, level of education, smoking status, residence and SARS-CoV-2 Positive test; ***p < 0.001. **p < 0.01; *p < 0.05.

## Discussion

Vitamin D deficiency has been associated with many adverse health outcomes, including cardiometabolic diseases. However, the prevalence of vitamin D deficiency among South African adults is not well established. For instance, there is no prior study on vitamin D from the Eastern Cape, a province with about 6.5 million people, which limits our understanding of nutritional deficiency in the country. Such data would enrich the South African literature on vitamin D status of the population and potentially, could inform local guidelines on vitamin D deficiencies. Therefore, the current study reports on the prevalence of vitamin D deficiency and its associations with cardiometabolic risk factors among residents of the Eastern Cape Province.

Serum 25(OH)D is the best marker for assessing vitamin D status, with individuals presenting a concentration range below 75 nmol/L considered vitamin D deficient^24^. In the current study, vitamin D deficiency was observed in 28.5% of the participants. The figure reported in this study is comparable to the 27% reported by Chutterpaul et al.^15^ among adult resident of KwaZulu-Natal, South Africa. The figure presented in this study is lower than the 31.3% reported in Northwestern Nigeria and 43.6% reported in Ghana^16,31^; however, it is higher than the 17.6% reported in Kenya^32^. Studies conducted in other parts of the world showed a higher prevalence of vitamin D deficiency than in the current study; United States of America (41.6%), Pakistan (73%), India (67%), Iran (90.7%) and Switzerland (51%)^17,33,34^. Similarly, the prevalence of vitamin D deficiency presented in this study was lower than the 83% reported in Fulani women (Nigeria)^35^ and the 46% reported by Naidoo et al.^36^ among South African women in KwaZulu-Natal province. Of note, we defined vitamin D deficiency as serum concentration of 25(OH)D < 50 nmol/L (28.29), while some of the reference studies defined vitamin D deficiency as 25(OH)D < 75 nmol/L (or 30 ng/ml). There are two criteria used to define vitamin D status; however, this lack of standardisation in the cut-off values of these two criteria complicates efforts to synthesise reliable epidemiological data and makes it hard to make comparisons between studies over time. Thus, it is important to establish standardised cut-off values for circulating 25(OH)D concentrations to generate accurate information about the prevalence of vitamin D deficiency across different populations.

Furthermore, we observed a higher prevalence of vitamin D deficiency among women (28.7%) than among men. Similar trends were also reported by Hovsepian et al.^37^, Bhutia et al.^38^ and Park et al.^39^. Furthermore, many studies suggest that vitamin D status is sex-related, although little is known regarding this association^40–42^. Therefore, we investigated the possible association between sex and vitamin D status. Although, vitamin D deficiency was common among women, we found no clear association between vitamin D status and sex. Results of the current study contradict findings reported by Muscogiuri et al.^42^ and Yan et al.^43^, where sex was a predictive factors of 25(OH)D concentrations. It was further demonstrated that the female sex was significantly associated with lower vitamin D levels than in men, and that female sex was independently associated with severe vitamin D deficiency^44^. On the other hand, Johnson et al.^45^ showed that the male sex was significantly associated with vitamin D deficiency in a Norwegian population. Based on these reports, it is not clear whether vitamin D status is affected by sex. The conflicting findings may be explained by varying study conditions, including differences in latitudes, age, sun exposure and body composition. It should also be noted that the current study is largely dominated by female participants (81.8%), thus, precluding full understanding of vitamin D status in the male population. More studies are needed to clarify the association between sex and vitamin D status.

In addition, circulating levels 25(OH)D are known to vary by race/ethnicity and age^46^. In the current study, vitamin D deficiency was associated with age. Our analysis further revealed that individuals who were ≥ 42 years old were more likely to be deficient in vitamin D. It is important to note that a large proportion (47.6%) of our study participants were aged between 46 and 65 years. Our findings differ from previous reports from the United Kingdom, where advanced age was associated with lower odds of vitamin D deficiency or insufficiency^47^. However, similar to a report made in the United States of America, where middle-aged and older adults had higher odds of vitamin D deficiency^48^. In addition, the current study revealed that vitamin D deficiency was common among black participants (29.9%). Our multivariate logistic regression analysis further demonstrated that black participants were more likely to exhibit vitamin D deficiency than other racial groups. Similar trends were reported by Meltzer et al.^49^, who found that low vitamin D levels were more common among black participants (36%) than among white participants (16%). Using data from the US National Health and Nutrition Examination Survey (NHANES), Weishaar et al.^50^ showed that darker skin colours were independently and significantly associated with poorer vitamin D status. Peiris et al.^51^ further demonstrated that the differences observed in vitamin D deficiency between black and white individuals were independent of latitude and seasonal changes. Data from the NHANES III also showed that 53%–76% of non-Hispanic blacks, compared with 8–33% of non-Hispanic whites, had 25(OH)D concentrations below 50 nmol/L in the winter^52^. In this study, seasonal changes of vitamin D concentrations were not assessed.

The higher prevalence of low vitamin D levels among black adults may be a result of lower cutaneous vitamin D synthesis attributable to higher melanin levels^53,54^ as well as lower intake of dairy products and other foods fortified with vitamin D. it should be noted that this study was carried out among the healthcare workers who are socioeconomically well off than the average people of the Eastern Cape province. As such, the true prevalence of vitamin D deficiency at the population level is expected to be much higher than the 28.5%. The findings of the current study, and others, warrant further studies on the potential role of vitamin D supplementation among black Africans.

In addition to ethnicity, we investigated the prevalence of DM, obesity, hypertension, CKD and metabolic syndrome among participants who met the criteria for vitamin D deficiency. We found a high prevalence of obesity (64.5%) among the study participants. Furthermore, the study showed that the prevalence of DM, hypertension, CKD, and metabolic syndrome ranged from 2.5 to 18.2% among participants with vitamin D deficiency. Our findings are in line with previous studies, in which cardiometabolic risk factors were prevalent among individuals who exhibited vitamin D deficiency^16,34,42,44^. However, our study found no association between vitamin D status and the selected cardiometabolic risk factors. In contrast, an analysis of data from the NHANES III 1988–1994 study showed that low vitamin D was significantly associated with CVDs and select CVD risk factors, including DM, obesity and hypertriglyceridemia^55^. A study conducted in South India demonstrated a strong association between hypertension and vitamin D deficiency^56^. On the other hand, in vivo studies show that low vitamin D levels are associated with increased systolic blood pressure accompanied by an increase in angiotensin 2, which is an important component of blood pressure regulation^55^.

In addition, Satirapoj et al.^57^ demonstrated that low vitamin D levels were associated with the level of kidney function, and Al-Dabhani et al.^58^ showed that vitamin D deficiency was associated with metabolic syndrome. It was further demonstrated that high levels of vitamin D were associated with a lower prevalence of metabolic syndrome^59^. Nonetheless, other studies have failed to confirm these observations^60,61^. Thus, future studies should investigate the causality between vitamin D status and known cardiometabolic risk factors.

## Study limitations

To the best of our knowledge, this is the first study to investigate the status of vitamin D, and also, its associated cardiometabolic risk factors among adult residents of the Eastern Cape province, South Africa. However, our study has several limitations that are worth mentioning. This study is limited to urbanised healthcare workers, who are gainfully employed by the state and therefore the status of vitamin D in the general population may be much worse. As such, a larger observational study targeting the broader population of Eastern Cape residents would elucidate the status of vitamin D in the region. Given the small number of minority ethnic groups participants included in this study, it is therefore difficult to establish the vitamin D status of this sub-population. Future studies should aim at recruiting a representative sample of minority populations to gain better understanding of vitamin D status in the country. With the limited number of male participants in the study, it is difficult to make definitive assessments about the vitamin D status within this particular subgroup. It is worth noting that the relatively low number of male participants in this study mirrors the gender distribution within the healthcare sector in South Africa, where women predominantly occupy roles such as nurses, cleaners, and other support staff. In addition, we did not assess diet, vitamin D supplements, parathyroid hormone levels as well as fasting blood sugar and lipid levels, which are important factors related to circulating serum 25(OH)D and metabolic syndrome. Second, we were not able to measure the lipid profile (triglyceride, HDL-C and LDL-C), blood glucose, blood pressure, waist circumference and seasonal variations of vitamin D levels. Although, we adjusted for potential confounders, this study has an observational design; therefore, residual confounding factors may remain. Nevertheless, the study could form a foundation for further studies on vitamin D status in poor regions of South Africa.

## Conclusion

In the current study, vitamin D deficiency was highly prevalent, particularly among women. Furthermore, the study confirmed the association between vitamin D status, ethnicity, and age. However, none of the selected cardiometabolic risk factors showed an association with vitamin D status. Additional data is needed to confirm the lack of association between these factors and vitamin D status. Nonetheless, the study highlighted the need for standardised cut-off values for 25(OH)D concentrations to generate reliable and representative national epidemiological data which will help to determine the incidence of vitamin D deficiency in the country. Furthermore, the findings of this study as well as those from previous reports highlight the need for intervention studies on the potential role of vitamin D supplementation among individuals of African origin.

## Data Availability

The dataset analysed in this study are available with the corresponding author upon reasonable written request.

## Abbreviations

BMI: Body mass index
CKD: Chronic kidney disease
CVDs: Cardiovascular diseases
DM: Diabetes mellitus
HCWs: Healthcare workers
HPLC: High performance liquid chromatography
NCDs: Non-communicable diseases
SARS-CoV-2: Severe acute respiratory syndrome coronavirus-2
WHO: World Health Organisation
